# AGE DIFFERENCES AND CHANGE IN COPING STRATEGIES ACROSS 10 YEARS: FINDINGS FROM THE MIDUS STUDY

**DOI:** 10.1093/geroni/igae098.2104

**Published:** 2024-12-31

**Authors:** Maria Kurth, Dakota Witzel, Eric Cerino, David Almeida

**Affiliations:** The Pennsylvania State University, University Park, Pennsylvania, United States; South Dakota State University, Brookings, South Dakota, United States; Northern Arizona University, Flagstaff, Arizona, United States; Pennsylvania State University, University Park, Pennsylvania, United States

## Abstract

Although age differences in coping strategies across adulthood are well-established (Aldwin et al., 2021), little is known about how coping strategies change, especially into later life. Further, studies focused on changes in coping relied on convenience samples (e.g., Brennan et al., 2012; Martin et al., 2008), which may not generalize to all midlife and older adults. Using data from waves two and three of the Midlife in the United States study, we examined (1) how coping changed over 10 years and (2) age differences therein. Participants (N = 2661, Mage_waveII = 54.56, age range: 30-84, 58% women, 87% White) completed 24 items from the COPE Inventory (Carver et al., 1989) at each wave. Four coping factors emerged from an exploratory factor analysis and confirmed via confirmatory factor analysis (Witzel & Kurth, 2023): instrumental action; denial/disengagement; positive reappraisal; focus/venting of emotions. Invariance was established across time and age. Two-level multilevel models, conducted separately for each coping strategy, revealed a significant decrease across 10 years for all strategies, even after controlling for race, gender, education, and sample type (core, Milwaukee). Older participants, compared to younger participants, decreased in their use of instrumental action and positive reappraisal, while denial/disengagement strategies increased; age did not moderate focus/venting of emotions. Preliminary evidence suggested non-linear changes by age for denial/disengagement and positive reappraisal. Discussion will surround the variability in how different types of coping changes across midlife and into older adulthood, and ways in which the rate and direction of this change depends on age.

